# High‐Dose L‐Serine Supplementation During Febrile Decompensation in *SARS1* Deficiency: A Case Report and Review of the Literature

**DOI:** 10.1002/jmd2.70090

**Published:** 2026-05-18

**Authors:** Victor Andrès Valle, Arnaud Wiedemnan, Eva Feigerlova, David Coelho, Elise Jeannesson, Irina Rotaru, Marc Merten, Alexandre Raharimanana, François Feillet, Jean‐Marie Ravel

**Affiliations:** ^1^ Université de Lorraine, CHRU‐Nancy, Reference Centre of Inborn Metabolism Diseases Nancy France; ^2^ Université de Lorraine, Inserm, NGERE Nancy France; ^3^ Laboratoire de biochimie, CHRU de Nancy Nancy France; ^4^ Laboratoire de génétique médicale, CHRU de Nancy Nancy France

**Keywords:** aminoacyl‐tRNA synthetase, cardiomyopathy, fever‐induced crisis, L‐serine, metabolic decompensation, rare inherited disorder, SARS1 deficiency

## Abstract

Seryl‐tRNA synthetase 1 (*SARS1*) deficiency is a rare autosomal recessive disorder presenting with neurodevelopmental delay, deafness, cardiomyopathy, and fatal metabolic decompensation triggered by febrile episodes. While amino acid chronic supplementation is established, no guidelines exist for acute management. We report the case of a 9‐year‐old male of Turkish origin with genetically confirmed *SARS1* deficiency, admitted with fever, vomiting, hypotonia, and seizures. The clinical course rapidly progressed to metabolic decompensation and severe acute cardiac failure, characterised by a left ventricular ejection fraction of 20%, necessitating mechanical ventilation and vasopressor support. Notably, the patient's family history included the death of three siblings during similar febrile episodes. During hospitalisation, the patient's specific L‐serine supplementation dosage was progressively tripled concurrently with standard supportive care. Unlike the fatal outcomes observed in his siblings, untreated by L‐serine, the patient survived and recovered following this high‐dose regimen. Cardiac biomarkers normalised within 20 days, and follow‐up echocardiography at 1 month demonstrated complete resolution of myocardial oedema. However, a year later, the patient presented with another febrile crisis at 10 years old, and despite an emergency protocol, the patient developed severe biventricular dysfunction progressing to fatal cardiogenic shock. This constitutes the first documented survival of a SARS1‐related metabolic crisis managed with high‐dose L‐serine. The findings strongly suggest that early, aggressive escalation of L‐serine dosage can be a viable therapeutic strategy for acute decompensation in SARS1 deficiency.

## Highlights

This case reports the first documented survival of acute metabolic decompensation in *SARS1* deficiency, achieved by tripling the patient's baseline L‐serine supplementation alongside standard supportive care during a febrile crisis. The outcome, contrasted with the fatal course in three untreated siblings, supports early aggressive L‐serine escalation as a potentially life‐saving strategy in this ultra‐rare disorder.

## 1 Introduction

Aminoacyl‐tRNA synthetases (aaRS) are essential enzymes that catalyse the attachment of amino acids to their cognate tRNAs, a prerequisite for accurate protein synthesis. Among them, seryl‐tRNA synthetase 1 (SARS1) catalyses the binding of serine to tRNASer and participates in protein synthesis [1]. Variants in aaRS genes are associated with a wide range of human disorders involving neurological, muscular, and metabolic dysfunctions [2, 3, 4, 5].

*SARS1* deficiency was first reported by Musante et al. [4] and later expanded by Ravel et al., who described a consanguineous Turkish family with a homozygous p.(Arg213Leu) variant leading to neurodevelopmental delay, deafness, hypertrophic cardiomyopathy, and fatal decompensation during febrile episodes [6]. *SARS1* was subsequently associated with autosomal recessive microcephaly [7], autosomal recessive spastic paraplegia [8] and autosomal dominant Charcot–Marie–Tooth disease [9].

The management of acute decompensation in *SARS1* deficiency remains undefined. Experimental data from related aaRS deficiencies suggest that supplementation with the cognate amino acid may enhance residual enzyme activity or serine incorporation (with the same enzyme) [10]. Here, we report the first case of survival following an acute febrile decompensation in a *SARS1* deficient patient treated with high‐dose L‐serine supplementation.

## 2 Case

The patient was a nine‐year‐old boy of Turkish origin, born to first‐cousin parents, previously diagnosed with *SARS1* deficiency harbouring a homozygous pathogenic variant c.638G>T p.(Arg213Leu) ([6], Figure S1).

He belonged to a family of five children, with four affected by a *SARS1* deficiency syndrome involving congenital deafness and developmental delays. The condition had severe manifestations in this family, with febrile episodes triggering catastrophic neurological events and death in three of the four affected children. The older brother had hypertrophic cardiomyopathy and died at 4 years old from sepsis that led to fatal cardiac failure. The second and the third siblings (twin sisters) died at 30 months and 9 years old after a febrile episode inducing status epilepticus, progressing to brain death within 48 h despite anticonvulsant therapy.

The reported patient, a 9‐year‐old boy, presented with similar symptoms (deafness, microcephaly and developmental delay). After the definitive molecular diagnosis, following his sibling's deaths, L‐serine supplementation was initiated at 100 mg/kg/day. He suffered from numerous episodes of hyperthermia with several ICU hospitalisations. At 9 years old, he had a febrile episode due to Influenza A confirmed by the ELISA test. Clinically, he presented with vomiting, hypotonia, and generalised seizures. Considering the fatal evolution of the siblings in similar conditions, the child was admitted to the intensive care unit. On admission, EEG showed parietal spikes, and lacosamide antiepileptic therapy was initiated. L‐serine supplementation was first doubled (200 mg/kg/day), then tripled (300 mg/kg/day), following deterioration (Table S1).

By day four, he developed acute respiratory distress syndrome due to pulmonary oedema (confirmed by chest X‐ray). Despite high‐flow oxygen, mechanical ventilation was required. Echocardiography revealed a severe drop in left ventricular ejection fraction (LVEF 20%). At Day 5, cardiac biomarkers confirmed myocardial injury (Table S2): troponin 6961 pg/mL (< 53 pg/mL) and NT‐proBNP 32 938 pg/mL (< 125 ng/L). Considering the hypothesis of myocarditis, a treatment with methylprednisolone and immunoglobulins was initiated.

**FIGURE 1:**
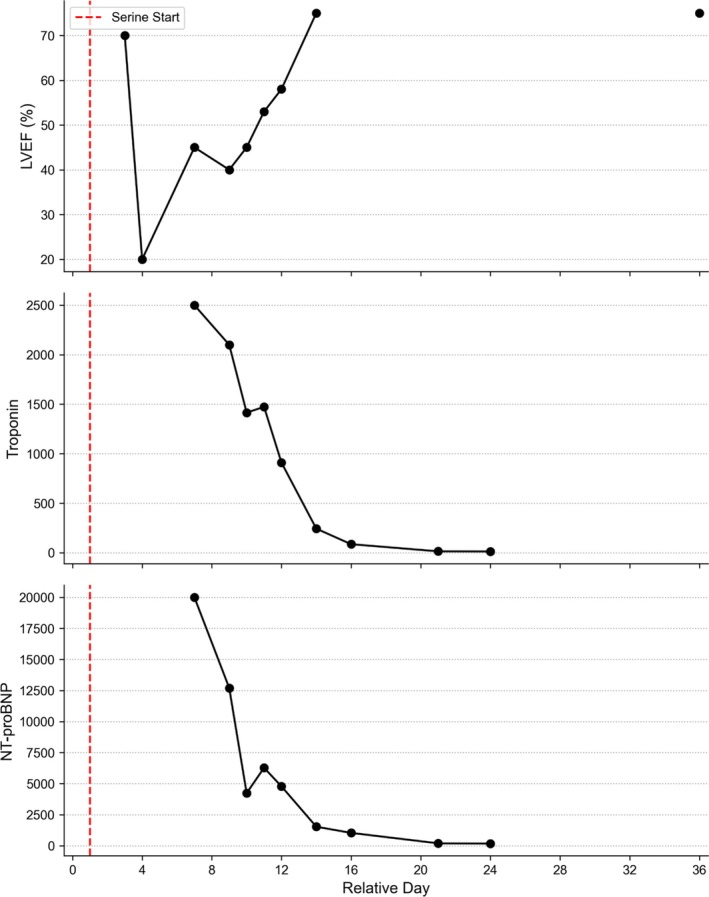
Cardiac function evolution during the acute febrile episode after L‐serine supplementation.

During this period, high‐dose L‐serine (300 mg/kg/day) was maintained through a nasogastric route. Progressive improvement was noted from Day 6 (LVEF 40%) to Day 14 (LVEF 70%, Figure 1). Biomarkers normalized by Day 20, and echocardiography at 1 month showed complete resolution of myocardial edema and cardiac function.

Baseline EEG showed a well‐organised, reactive background rhythm of 9–10 Hz (normal for age), but with abnormalities identified as right temporo‐parietal spikes. Following the acute event, the background rhythm slowed significantly to 5–6 Hz, indicating an acute encephalopathic impairment. However, at this stage, there were no epileptic patterns (spikes or spike‐waves). The background rhythm during the recovery phase remained slow (5–6 Hz), but focal abnormalities reappeared with parietal spikes. A brief, one‐second burst of bilateral spike‐waves was noted without any clinical correlation. Unlike his siblings, the patient's EEG did not evolve toward electrocerebral silence. Instead, this monitoring captured a transient encephalopathy that gradually resolved.

During the patient's stay in the paediatric ICU, a significant and transient hyperlactatemia was observed (Table S3). Whilst initial venous lactate was only slightly elevated (1.8 mmol/L), a severe lactic acidosis developed concurrently with an acute episode of respiratory failure. Critically, this metabolic derangement was rapidly reversible, with levels normalising within 24 h of the peak (1.2 mmol/L arterial, 0.9 mmol/L venous). Following one subsequent, milder elevation (1.9 mmol/L venous), lactate levels stabilised within the normal range.

A year later, the patient presented another crisis at 10 years old. Emergency protocol with increased L‐Serine dose was instituted. Nonetheless, he had to be transferred from home to our P.I.C.U. because of his altered mental status. A slowed background rhythm on his EEG was consistent with metabolic encephalopathy. The doses of L‐Serine were increased to 500 mg/kg/day (5 times the usual dosage), leading to cognitive improvement. Despite this initial improvement, he presented with a severe biventricular dysfunction, progressing to cardiogenic shock despite vasopressor support (dobutamine up to 20 mcg/kg/min) and mechanical ventilation. He developed near‐complete akinesia of cardiac function, associated with pericardial effusion. Neurological reassessment revealed fixed and dilated pupils, and death was subsequently pronounced.

## 3 Discussion

This report presents the first known survival of a *SARS1* deficient patient following a severe febrile decompensation, contrasting with the previously fatal outcomes described in the brother and sisters of this patient by Ravel et al. [6]. The most notable difference in management was the early and sustained tripling of L‐serine supplementation, suggesting a potential therapeutic benefit of increasing the dosage of L‐serine during intercurrent infections in these patients.

In vitro data indicate that SARS1 enzyme activity is temperature‐sensitive [6, 10]. During fever, enzymatic instability may exacerbate serine deficiency in protein synthesis and protein production, precipitating metabolic crisis. L‐serine supplementation may counteract this effect by saturating residual enzymatic activity, as previously proposed for other aaRS deficiencies such as MARS1 and LARS1 [10].

The patient's rapid cardiac recovery further supports a role for serine metabolism in maintaining myocardial function under stress. While corticosteroids and immunoglobulins may have contributed, the parallel normalisation of troponin levels and LVEF following serine escalation strengthens the hypothesis of metabolic rescue.

The pathophysiology of fatal febrile decompensation in patients with the constitutional *SARS1* p.(Arg213Leu) likely pathogenic variant seems best explained by a “saturation failure” model rather than simple protein thermolability [10]. The p.(Arg213Leu) variant establishes a chronic, sub‐optimal ceiling on cytoplasmic serylation capacity [6]. This baseline deficit creates a persistent bottleneck in the translation of nuclear DNA‐encoded mitochondrial proteins, thereby compromising basal OXPHOS maintenance. Fever acts as a potent dual accelerator in this vulnerable state. First, it drives hypermetabolism, increasing systemic ATP demand. Second, it induces significant translational competition; the limited seryl‐tRNA pool is critically diverted away from mitochondrial protein synthesis and toward the acute synthesis of stress‐response proteins, such as cytokines. This acute diversion completely overwhelms the already limited serylation capacity, precipitating a total collapse of mitochondrial ATP production. This acute failure of energy metabolism manifests as catastrophic, high‐energy‐demand organ failure, including intractable status epilepticus and fatal cardiac akinesia.

Another hypothesis to consider is the potential toxicity of L‐serine supplementation itself, particularly given the high‐dose regimen initiated. However, this possibility appears unlikely based on the robust safety profile established in both clinical and preclinical studies. High‐dose L‐serine has been evaluated in several human trials without reports of significant organ damage. For instance, a Phase I trial in patients with Amyotrophic Lateral Sclerosis (ALS) demonstrated that doses up to 30 g/day (15 g twice daily) administered for 6 months were generally well‐tolerated [11]. Similarly, a 48‐week study utilizing high‐dose L‐serine (approximately 15–30 g/day) in patients with Hereditary Sensory and Autonomic Neuropathy type 1 (HSAN1) reported no serious adverse events [12]. This strong clinical safety profile is consistent with preclinical data, as subchronic toxicity studies in rats have also failed to identify any marked toxicity associated with L‐serine [13].

The patient experienced a fatal crisis a year later despite the immediate initiation of an emergency protocol. It remains unclear exactly why the latest, higher dose failed. Potential variables that may have influenced this outcome include differences in the specific infectious etiologies (A flu in the first crisis and B flu in the fatal one). Furthermore, this treatment failure suggests that the proposed “saturation failure” mechanism may have a physiological limit. If the systemic ATP demand and translational competition induced by hyperthermia become too extreme, the diversion of the seryl‐tRNA pool may completely overwhelm mitochondrial protein synthesis, rendering even massive substrate saturation futile. Therefore, while this case initially supports the potential benefit of prompt L‐serine escalation, the subsequent fatal outcome underscores the profound severity of SARS1 deficiency and indicates that L‐serine supplementation, even at extreme doses, is not a guaranteed rescue for every episode of febrile decompensation.

As a single‐case observation, causality cannot be conclusively established. Controlled studies are required to determine optimal dosing, timing, and long‐term outcomes. Future research should focus on developing standardised L‐serine emergency protocols for *SARS1* related crises, identifying biochemical markers of serine metabolism during febrile episodes, and assessing whether continuous high‐dose supplementation can prevent metabolic decompensation.

This case supports the potential benefit of high‐dose L‐serine supplementation in managing febrile metabolic decompensation in *SARS1* deficiency. Prompt serine escalation during crisis may prevent fatal outcomes and warrants further investigation as a targeted therapy for this ultra‐rare disorder. These patients require an emergency protocol with triple supplementation in the event of fever, and only time will tell whether this is truly beneficial.

## Funding

The authors have nothing to report.

## Consent

Informed consent was obtained from the parents.

## Conflicts of Interest

The authors declare no conflicts of interest.

## Supporting information


**Figure S1:** Pedigree of the reported case. Adapted from Figure S1, Ravel et al. [1].

**Table S1:** Timetable of serine supplementation.

**Table S2:** Evolution of general clinical parameters, cardiac function, and laboratory biomarkers during the pediatric intensive care unit (PICU) hospitalization. This table details the daily progression of the patient's vital signs, life‐support interventions (including mechanical ventilation and vasopressor use), and metabolic markers during the first severe acute febrile episode (at 9 years of age). The timeline demonstrates the temporal relationship between the escalation of L‐serine supplementation (initially doubled to 200 mg/kg/day, then tripled to 300 mg/kg/day) and the patient's clinical recovery. Specifically, it tracks the acute drop and subsequent recovery of the Left Ventricular Ejection Fraction (LVEF), which improved from a nadir of 20% to 70% by Day 14. Cardiac injury is reflected by peak values at Day 5, with subsequent normalization tracking alongside the L‐serine dose increase. EEG, Electroencephalogram; LVEF, left ventricular ejection fraction; NT‐proBNP, N‐terminal pro b‐type natriuretic peptide; PICU, Pediatric Intensive Care Unit.

**Table S3:** Evolution of blood gas and metabolic parameters during the acute decompensation episode. This table details the daily progression of the patient's arterial and venous blood gas values, electrolytes, and L‐lactate levels over the course of hospitalization. It specifically highlights the severe and rapid onset of hyperlactatemia (peaking at a venous L‐lactate of 9.9 mmol/L on Day 4), which developed concurrently with acute respiratory failure. Following intubation, vasopressor support, and the escalation of L‐serine supplementation, this severe lactic acidosis proved to be rapidly reversible, with levels normalizing within 24–48 h. A subsequent, milder elevation in venous lactate (1.9 mmol/L) was noted on Day 8 before stabilizing completely within normal limits. pCO_2_, partial pressure of carbon dioxide; pO_2_, partial pressure of oxygen.

## Data Availability

The data that support the findings of this study are available from the corresponding author upon reasonable request.
