# Supplementary figures and images for: High‐Dose L‐Serine Supplementation During Febrile Decompensation in SARS1 Deficiency: A Case Report and Review of the Literature
Source: JIMD Rep. 2026 May 18;67(3):e70090. doi: 10.1002/jmd2.70090 (PMC13181329; doi:10.1002/jmd2.70090)

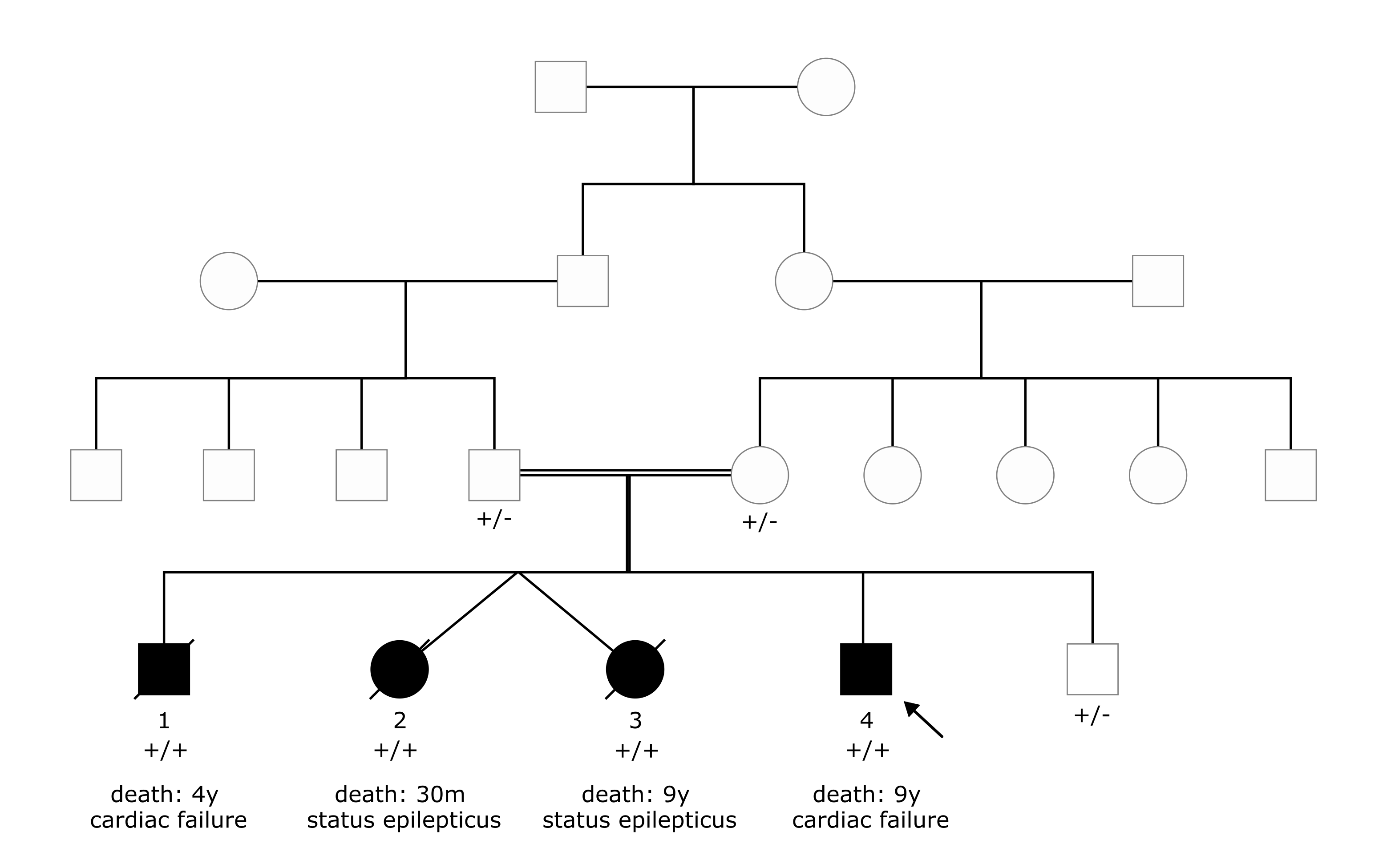

Supplement: Supplementary file 1 — Figure S1: Pedigree of the reported case. Adapted from Figure S1, Ravel et al. [1]. [file JMD2-67-e70090-s004.png]
